# Angiogenic microRNAs in Systemic Sclerosis: Insights into Microvascular Dysfunction and Therapeutic Implications

**DOI:** 10.3390/genes16091057

**Published:** 2025-09-09

**Authors:** Marta Rusek

**Affiliations:** Independent Unit of Radiopharmacy, Department of Organic Chemistry, Faculty of Pharmacy, Medical University, 4a Chodźki Street, 20-093 Lublin, Poland; marta.rusek@umlub.edu.pl

**Keywords:** systemic sclerosis, microRNAs, angiogenesis, endothelial dysfunction, biomarkers, fibrosis, epigenetics, therapeutic targets

## Abstract

Systemic sclerosis (SSc) is a complex connective tissue disease that affects the skin and internal organs and is characterized by immune dysregulation, progressive fibrosis, and microvascular dysfunction. Chronic tissue ischemia, accompanied by impaired angiogenesis, leads to the gradual loss of small vessels, resulting in clinical complications, such as Raynaud’s phenomenon, digital ulcers, pulmonary arterial hypertension, and renal crisis. Emerging evidence highlights the crucial regulatory role of microRNAs (miRNAs) in vascular homeostasis through the modulation of key signaling pathways and endothelial cell activity. Dysregulated miRNAs influence fibroblast proliferation, inflammatory responses, and immune cell activity in SSc, contributing to disease progression. Current knowledge is still limited, highlighting the need for further research to elucidate the miRNAs network involved in the etiopathogenesis of SSc. The use of miRNA-based biomarkers is gaining tremendous attention for early diagnosis, risk stratification, classification, and the prediction of therapeutic responses. This review provides insights into angiogenesis-related miRNAs involved in SSc pathogenesis, discusses their relevance as biomarkers, and explores their promise as therapeutic targets. Advancing our knowledge of miRNAs-mediated regulatory networks may open new possibilities for personalized approaches to SSc management.

## 1. Introduction

Systemic sclerosis (SSc) is a rare autoimmune disease with an unknown underlying cause [1], characterized by immune system disorders, microvasculopathy, and excessive collagen deposition in the skin and internal organs, such as the lungs, kidneys, gastrointestinal tract, and heart [2,3,4]. The clinical manifestations are heterogeneous among patients; thus, SSc is classified as limited or diffuse, based on the distribution of cutaneous involvement [3].

Microvascular dysfunction is recognized as one of the earliest pathogenic events in SSc, often preceding fibrotic remodeling. Capillary narrowing, endothelial apoptosis, and rarefaction of the microvasculature contribute to tissue hypoxia and affect the repair processes [5]. It is driven by chronic endothelial injury, impaired differentiation of endothelial progenitor cells (EPCs), and aberrant signaling by angiogenic factors, such as vascular endothelial growth factor (VEGF) and endothelin-1 (ET-1) [6,7]. Therefore, there is increased adhesion of peripheral monocytes, macrophage polarization toward the M2 phenotype (also known as alternatively activated macrophages, CD206^+^/ARG1^+^) with profibrotic properties, and macrophage-to-myofibroblast transition in the skin and affected organs in SSc [4,8]. The final consequence is fibrosis through multiple signaling pathways [9,10].

The disease course is unpredictable, ranging from a slowly evolving disease to rapid multi-organ deterioration. Several clinical cohort studies of SSc have been conducted in many countries worldwide to determine the clinical features and disease progression [11,12]. Noteworthy, autoantibodies against intracellular antigens are associated with the specific clinical features of the disease. In contrast, autoantibodies against cell surface antigens may induce endothelial cell (ECs) injury, which is widely recognized as a key initiating event in the pathogenesis of the disease [13,14].

Although genetic predisposition and environmental exposure have been implicated, the precise mechanisms underlying the pathogenesis of SSc remain uncertain. Increasing evidence points to a role of epigenetic regulation of gene expression, including alterations in DNA methylation, changes in chromatin structure, histone modification, and miRNA expression, in modulating immune responses and fibrosis [15,16]. Among these, miRNAs are of particular interest because they affect gene expression and may serve as both biomarkers and therapeutic targets [17,18,19]. Since SSc complications significantly impact the quality of life, future research should focus primarily on the early phases of angiogenesis disturbances, which are dominated by the excessive presence of pro-angiogenic factors and microvascular changes [20,21]. The initial ECs injury can be induced by various factors, including environmental factors, viral infections, anti-endothelial cell antibodies (AECAs), ischemia-reperfusion events, and activity of reactive oxygen species (ROS) [22]. This review aims to outline the current understanding of the contribution of angiogenesis-related miRNAs to vascular pathology in SSc, with an emphasis on their biological roles, clinical relevance, and potential applications for early diagnosis and treatment.

## 2. microRNAs Biogenesis and Function

Recent studies highlight miRNAs as central epigenetic regulators in SSc, modulating gene networks involved in immune response, fibrosis, and angiogenesis. Thus, miRNAs have been considered potential disease biomarkers and drug targets [23,24]. MiRNAs are short, non-coding RNA sequences, generally 19–25 nucleotides in length, that function as post-transcriptional regulators of gene expression by suppressing specific target mRNAs. They exert their function mainly by pairing to the 3′-untranslated region (3′-UTR) of messenger RNAs (mRNAs), which results in either suppression of translation or degradation of the transcript. Thus, miRNAs influence diverse cellular processes, including proliferation, differentiation, apoptosis, and immune regulation [25].

The biosynthesis of mature miRNAs is a multistep process that involves both nuclear and cytoplasmic events. In the nucleus, miRNA genes are transcribed primarily by RNA polymerase II to form long transcripts, known as primary miRNAs (pri-miRNAs). Subsequently, the RNase type III enzyme Drosha, in association with its cofactor, double-stranded RNA-binding protein (DGCR8), processes these transcripts into hairpin-shaped molecules of approximately 70 nucleotides, known as precursor miRNAs (pre-miRNAs) [25,26,27].

The pre-miRNAs are then recognized by exportin 5 (XPO5), which transports them through the nuclear pore into the cytoplasm in a RanGTP-dependent manner [21].

**Figure 1:**
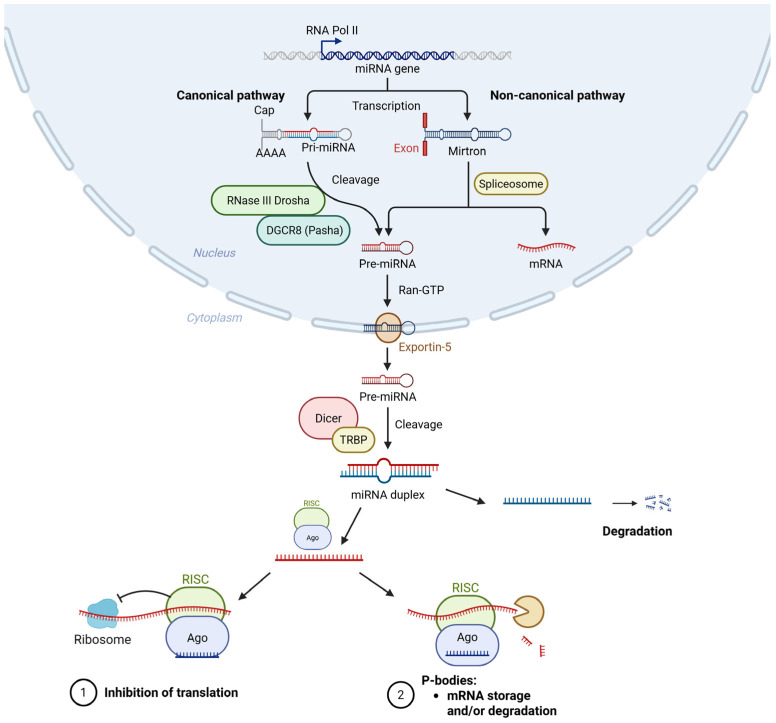
Biogenesis of miRNAs. The biogenesis of miRNAs starts in the nucleus, where RNA polymerase II transcribes pri-miRNAs. These are cleaved by the Drosha-DGCR8 complex into ~70-nt pre-miRNAs, which are transported to the cytoplasm by exportin-5. There, Dicer further processes pre-miRNAs into short miRNAs duplexes, from which one strand is loaded into the RISC to regulate target mRNAs. Based on [25,26]. Created in BioRender. Rusek, M. (2025). Available online: https://BioRender.com/kz5oa5w (accessed on 24 July 2025).

Once exported into the cytoplasm, the RNase III endonuclease Dicer processes the pre-miRNA to generate duplex-miRNAs (ds-miRNAs). This molecule typically contains a phosphate at the 5′ end and at the 3′ end, a two-nucleotide overhang with a hydroxyl group [25,27]. One strand of this duplex, termed the guide strand, is incorporated into the RNA-induced silencing complex (RISC), whereas the other strand is usually degraded [25,26]. Guided by the miRNA sequence, RISC binds to partially or fully complementary sites within the 3′ UTR of target mRNAs [28]. The level of complementarity typically determines the degree of transcriptional regulation; perfect complementarity leads to mRNA cleavage and decay, while imperfect pairing more commonly results in mRNA silencing through repression of translation or sequestration of the transcript [25,29]. An overview of the key processes involved in miRNA biogenesis is presented in Figure 1.

miRNAs control gene expression primarily through sequence-specific binding to complementary regions at the 3′-UTR of target mRNAs [27]. This interaction between miRNAs and mRNA typically suppresses gene expression either by inhibition of mRNA translation or by promoting degradation of transcript [27]. Thus, at the post-transcriptional level, miRNAs act as negative regulators of gene expression [27]. Enzymes that are essential for miRNAs biogenesis, such as Drosha and Dicer, are tightly regulated. Dicer processes diverse RNA classes, including tRNA and snoRNA, and maintains genome integrity. Alterations in their activity can affect the miRNA profile and their target genes [27]. Recent studies emphasize that dysregulation of miRNA biogenesis enzymes, such as Drosha and Dicer, may contribute to altered miRNA profiles in autoimmune diseases and fibrotic disorders, including SSc [4].

miRNAs are easily detected and stable in various biological sources, including tissues, serum, and other bodily fluids [30]. Therefore, miRNAs are attractive candidates for use as disease biomarkers [31]. As regulators of post-transcriptional gene expression, miRNAs play essential roles in numerous physiological and pathological processes, including differentiation, development, migration, proliferation, and apoptosis [30,32,33,34,35]. The expression profile of miRNAs reflects the underlying pathophysiological processes and has been linked to pathological conditions that involve the modulation of immunity [36,37], infectious and regenerative diseases, cardiac disorders [37], neurological disorders [38], and carcinogenesis [39]. Of note, miRNAs are not confined to intracellular compartments. They are also released into the extracellular environment, either encapsulated in exosomes and microvesicles or associated with proteins and lipoproteins [38].

In addition to their role in canonical gene silencing, miRNAs participate in extended post-transcriptional regulatory networks that include interactions with other non-coding RNAs, such as long non-coding RNAs (lncRNAs) and circular RNAs (circRNAs). Certain circRNAs (e.g., CDR1as/ciRS-7) exemplify potent miRNAs sequestration by acting as “miRNAs sponges”. However, most lncRNAs and circRNAs are expressed at levels too low to function as effective sponges; their influence on miRNAs activity is generally modest and highly context-dependent, operating within a broader competing endogenous RNAs (ceRNAs) network [40,41,42]. These interactions add another layer of complexity to post-transcriptional regulation and may expand the potential of miRNA-based diagnostics and therapeutics.

## 3. Endothelial Dysfunction and Microvascular Damage in SSc

One of the earliest hallmarks of SSc is vascular injury, which frequently occurs before fibrosis and immune activation [22]. The endothelium, the inner lining of blood vessels formed by ECs, plays a central role in regulation of angiogenesis and vascular tone and is strongly influenced by angiogenic factors [43]. In SSc, persistent endothelial injury leads to dysregulated angiogenesis, intimal proliferation, and rarefaction of the microvasculature, resulting in chronic tissue ischemia and organ dysfunction [22]. Microvasculopathy is characterized by an irregular and disorganized architecture of capillaries and small vessels and their loss [44]. Moreover, angiogenesis, including ECs migration and capillary formation, remains insufficient. Although pro-angiogenic signals are present, they are overwhelmed by endothelial injury, anti-angiogenic mediators, and a stiff fibrotic extracellular matrix (ECM), which impairs endothelial migration and capillary formation. Consequently, vascular repair fails, leading to progressive capillary rarefaction and ischemic complications [45]. This pathogenic mechanism results from an imbalance between angiogenic and angiostatic factors [46], including increased levels of VEGF, platelet-derived growth factor (PDGF), tumor growth factor (TGF-β), thrombospondin-1 (TSP1), and angiostatin, and decreased levels of nitric oxide (NO) and prostacyclin [47]. Thus, this imbalance contributes to the dysregulation of vascular tone and chronic inflammation and the promotion of fibrotic remodeling [44].

Microvascular endothelial cells (MVECs) are particularly susceptible to immune-mediated injury in SSc as they are key regulators of vascular homeostasis and angiogenesis [48]. Persistent injury to microvascular ECs contributes to the upregulation of adhesion molecules, leukocyte adhesion and transmigration, platelet activation, thickening of the basal membrane, and proliferation of surrounding pericytes, fibroblasts, and smooth muscle cells [49,50]. The forming perivascular infiltrate is composed of dendritic cells, B lymphocytes, and T lymphocytes [51], reflecting the contribution of adaptive immunity to the vascular pathology.

Chronic inflammation and endothelial apoptosis promote the opening of intercellular junctions and facilitate endothelial-to-mesenchymal transition (EndoMT), contributing to intimal hyperplasia, thickening of vessel walls, and progressive narrowing of the lumen [52]. EndoMT is a key mechanism in SSc vasculopathy, in which ECs lose their markers, such as CD31 and vascular endothelial cadherin (VE-cadherin), and acquire mesenchymal and myofibroblast features, such as α-smooth muscle actin (α-SMA) and vimentin [53]. EndoMT is driven by multiple stimuli, including TGF-β/Smad signaling, hypoxia, proinflammatory cytokines such as interleukin 1β (IL-1β) and tumor necrosis factor α (TNF-α), and oxidative stress [53]. Dysregulated miRNAs like miR-21, miR-155, and miR-200c have been implicated in the modulation of EndoMT, highlighting their potential as therapeutic targets [48,49,50]. Next, abortive reparative neoangiogenesis with abnormal capillary proliferation is also present [52,54]. Vascular manifestations can be observed early in patients with SSc. These include the initial derangement of capillaries, malformed capillaries, Raynaud’s phenomenon, and digital ulcers [52]. In later stages, it leads to fibroproliferative vascular lesions in multiple organs and may result in critical organ injuries, such as pulmonary arterial hypertension (PAH) and renal crisis [55]. The development of vasculopathy during SSc is associated with complex interactions between ECs, fibroblasts, lymphocytes, and macrophages, as well as the action of several cytokines, chemokines, and growth factors released by inflammatory and mesenchymal cells [22].

Recent studies have suggested that neovascularization impairment in SSc may be related to both angiogenesis and vasculogenesis failure [55]. Angiogenesis refers to the of new capillaries from preexisting blood vessels [56]. This process is balanced and regulated and involves ECs activation, proliferation, migration, and invasion into the extracellular space, followed by sprouting and lumen formation of capillaries, which is mainly triggered by tissue hypoxia or injury [56]. However, elevated levels of pro-angiogenic mediators are present in SSc, and the overexpression of angiostatic factors has also been observed [57]. Therefore, both angiogenesis and vasculogenesis are impaired in SSc. Angiogenesis, the sprouting of new capillaries from preexisting vessels, is initiated but fails to produce stable vascular networks. Vasculogenesis, the generation of new vessels from EPCs, is also defective due to impaired progenitor function and survival. Elevated VEGF levels, paradoxically, can drive the formation of aberrant vessels rather than effective microvascular repair. The progressive loss of capillaries is associated with high plasma levels of the VEGF [58].

Moreover, there is an imbalance in vasoactive factors, such as increased levels of ET-1 and decreased levels of NO and prostacyclin [59], affecting vascular tone, inflammation, and fibrosis [49,60]. VEGF plays a key role in the control of numerous cellular and molecular processes involved in the angiogenic cascade [21,60]. Indeed, it promotes ECs increasing their migration and beginning the proliferative process until a complete tubular structure is formed [21,59]. VEGF is a regulatory factor involved in angiogenesis and is overexpressed in both the skin and serum of patients with SSc [60]. Moreover, serum VEGF levels are correlated with the development of fingertip ulcers [60]. Although increased expression of VEGF induces the formation of new vessels, prolonged exposure leads to the formation of megacapillaries and chaotic vessel networks; thus, blood flow is reduced in patients with SSc [61]. 

**Figure 2:**
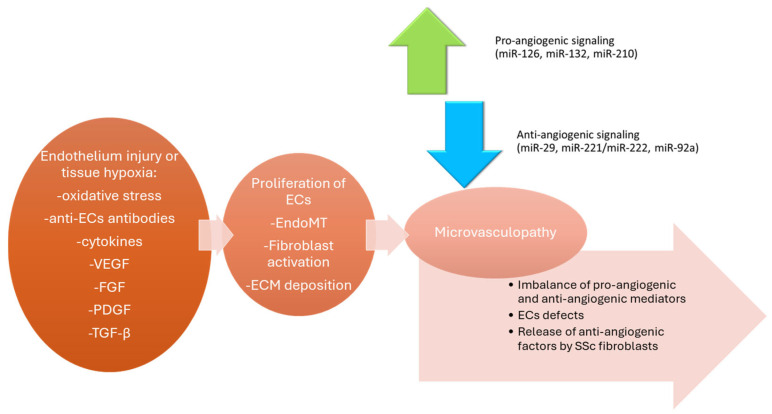
Schematic diagram showing the potential mechanisms involved in impaired neoangiogenesis in SSc. Based on [1]. ECs, endothelial cells; SSc, systemic sclerosis.

Additional contributors include dysfunctional circulating progenitors, fibroblast-derived anti-angiogenic mediators, and aberrant expression of transcription factors, such as Fos-related antigen-2 (Fra2) and Friend leukemia integration 1 transcription factor (Fli1), which may further contribute to SSc vasculopathy [62,63]. It is noteworthy that endothelial injury is a condition that could also be mediated by specific autoantibodies, such as anti-endothelial cell antibodies [44,64]. Potential mechanisms leading to impaired neoangiogenesis in SSc are presented in Figure 2.

Nailfold videocapillaroscopy (NVC) is a validated tool for the detection of early microvascular abnormalities in patients with SSc. Typical findings include dilated or distorted capillaries, loss of capillary density, avascular areas, and disorganized architecture [65,66,67,68]. Progressive capillary loss correlates with functional manifestations, such as Raynaud’s phenomenon, digital ulcers, and ischemic tissue damage [68,69]. Moreover, advanced techniques, such as optical coherence tomography angiography (OCTA) and contrast-enhanced ultrasound (CEUS), are increasingly used to assess microvascular perfusion and may be incorporated into future diagnostic algorithms [70,71,72]. Modern imaging tools are non-invasive tools to monitor microvascular pathology. Nevertheless, the molecular underpinnings of vascular injury in SSc remain incompletely understood, underscoring the need for mechanistic and translational research.

## 4. Angiogenic and Anti-Angiogenic miRNAs in SSc

MiRNAs are abundantly expressed within the cardiovascular system, where they regulate vascular homeostasis, remodeling, and repair [4]. In SSc, altered expression of specific miRNAs contributes to ECs injury, abnormal vessel growth, and impaired regenerative responses [73]. Some miRNAs enhance angiogenesis and vascular repair, while others inhibit these processes, favoring fibrosis and ischemia. The balance between these opposing groups of miRNAs is believed to influence the severity of vascular complications in SSc [56]. The inhibition of two endonucleases required for mature miRNA generation (Dicer and Drosha) *in vitro* and *in vivo* has established a potential importance for miRNAs in vascular development and ECs function [74]. Dysregulation of miRNAs can shift this balance toward either excessive or insufficient vessel formation, thereby exacerbating vascular pathology [75].

**Figure 3:**
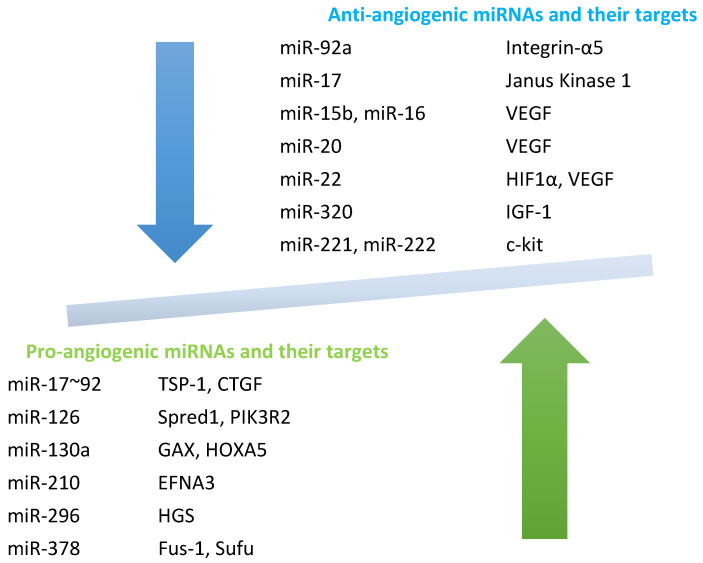
Potential target genes that may play a pro-angiogenic (stimulating) and/or anti-angiogenic (inhibiting) role in SSc pathogenesis. Based on [27,77,78].

In SSc, unique miRNAs profiles (also referred to as “angiomiRs”) have been identified. These miRNAs target major regulators of angiogenesis, including hypoxia-inducible factor 1α (HIF1α), cytokines, growth factors, such as epithelial growth factor (EGF)-like domain-containing protein 7 (EGFL7), fibroblast growth factor 11 (FGF11), platelet-derived growth factor receptor beta (PDGFRB), the vascular endothelial growth factor family, and metalloproteinases (MMPs) [76]. Depending on their activity, miRNAs can be broadly categorized as pro-angiogenic or anti-angiogenic, as shown in Figure 3, with potential target genes in SSc pathogenesis.

Pro-angiogenic miRNAs include miR-17~92 [79], miR-126 [80,81], miR-130a [82], miR-210 [83], miR-296 [84], and miR-378 [85]. In contrast, anti-angiogenic miRNAs include miR-92a [86], miRNA-17 [87], miR-15b, miR-16, miR-20a, miR-20b [88], miR-320 [89], miR-221 and miR-222 [90]. An overview of miRNAs that affect vasculopathy in SSc is presented in Table 1.

### 4.1. Pro-Angiogenic miRNAs

Several miRNAs promote angiogenesis by promoting ECs proliferation, survival, migration and suppression of the inhibitory pathways.

#### 4.1.1. miR-126

miR-126 is essential for preserving vascular integrity and homeostasis following tissue injury by enhancing ECs repair capacity [147]. Multiple studies have demonstrated that miR-126 regulates inflammatory cell recruitment, migration, capillary organization and network stability, angiogenic sprouting, and ECs survival by repression of negative regulators within the VEGF signaling pathway [73,81,98,138,148]. miR-126-5p facilitates ECs proliferation by suppressing the Notch1 inhibitor delta-like 1 homologue (Dlk1), thus increasing angiogenesis [149]. In contrast, miR-126-3p enhances ECs angiogenesis by suppressing negative regulators of the VEGF pathway, including sprouty-related protein-1 (SPRED1) and phosphoinositol-3 kinase regulatory subunit 2 (PIK3R2/p85-β) [149].

Although miR-126 is classically pro-angiogenic and its level is reduced in SSc MVECs with impaired VEGF responses [150], clinical cohorts have reported discordant circulating levels. In a recent case–control study, serum miR-126 was lower in SSc and was associated with PAH and pulmonary fibrosis [151], whereas another cohort found no significant difference compared to the control group [152]. Potential reasons include the sample type (tissue, serum, and extracellular vesicles), normalization strategies, and disease subset/severity.

Interestingly, miR-126 has an intronic location in the epithelial growth factor (EGF)-like domain-containing protein 7 (EGFL7) gene and contributes to the regulation of its transcription in ECs [97]. Alteration of the EGFL7/miR-126 regulatory axis has been described in SSc skin [153]. According to Harris et al., miR-126 downregulates vascular cell adhesion molecule 1 (VCAM1) expression, which is involved in leukocyte adhesion to the endothelium [98]. Thus, decreasing miR-126 level in ECs increases TNF-stimulated VCAM-1 1 expression and enhances leukocyte adherence to ECs [98]. Inhibition of miR-126 has been shown to increase TNF-α expression, which activates nuclear factor kappa beta (NFκB) and interferon regulatory factor 1, ultimately driving VCAM 1 expression and leukocytes adhesion to ECs [154].

Most studies are derived from developmental or cancer angiogenesis models rather than SSc-specific contexts. Clinical studies involve small patient cohorts, and the differential roles of the 3p and 5p isoforms complicate interpretation. In HUVECs and dermal microvascular ECs, miR-126-3p promotes endothelial proliferation and tube formation by repressing Spred-1 and PIK3R2, which are negative regulators of VEGF signaling [149]. Moreover, patient-derived SSc microvascular ECs show reduced miR-126 expression, correlating with increased leukocyte adhesion through VCAM-1 upregulation [84]. miR-126-5p enhances angiogenesis by suppressing the Notch1 inhibitor Dlk1 [149]. The angiogenic activity of miR-126 has been shown in zebrafish and mouse models with the knockdown of miR-126, causing impaired vascular integrity [93]. Thus, miR-126 modulates vascular inflammation by suppressing leukocyte adhesion to ECs [98]. Van Solingen et al. have shown that miR-126 contributes to arteriogenesis and angiogenesis because using a high dose of antagomir-126 significantly decreased the angiogenic response [138]. miR-126 is strongly pro-angiogenic in model systems, but its precise contribution to the vascular pathology of SSc remains incompletely validated.

#### 4.1.2. miR-130a

Another important miRNA involved in angiogenesis regulation is pro-angiogenic miR-130a [82]. It promotes vascular growth by suppressing anti-angiogenic homeobox proteins, including growth arrest homeobox (GAX) and homeobox protein HOX-A5 (HoxA5). GAX inhibits EC proliferation, migration, and tubulogenesis, while HoxA5 is a crucial regulator of tubule formation in ECs [147]. Direct evidence in SSc is lacking, and most data are derived from generic ECs cultures or tumor angiogenesis models.

#### 4.1.3. miR-210

Upregulation of miR-210 plays a central role in the ECs response to hypoxia [83]. Its overexpression promotes primary capillary network formation and enhances VEGF-induced cell migration, whereas miR-210 inhibition suppresses capillary formation, reduces migration, and induces apoptosis [83]. One of its key targets is ephrin (Eph)-A3, a ligand of Eph receptor tyrosine kinases, whose stimulation is essential for capillary-like structure formation and endothelial cell chemotaxis in response to VEGF and hypoxia [83,91]. There has been no direct validation in SSc-derived ECs or animal models; hypoxia in SSc vasculopathy may involve distinct pathways.

#### 4.1.4. miR-296

Würdinger et al. demonstrated that miR-296 is a critical mediator of angiogenesis and that up- or down-regulation leads to the induction and inhibition of morphologic features associated with ECs angiogenesis, respectively [84]. Moreover, inhibition of miR-296 using antagomirs significantly reduced angiogenesis in tumor xenografts *in vivo* [43,84]. Until now, there is no SSc-specific data; therefore, the role of miR-296 in SSc angiogenesis remains hypothetical.

#### 4.1.5. miR-148b

miR-148b is a crucial component of angiogenesis, acting through the regulation of target genes TGF-β2 and SMAD2 [155]. Its inhibition promotes the EndoMT process, while its overexpression enhances vascularization and promotes wound closure, whereas its suppression accelerates EndoMT in the wound and impaired healing [155]. Thus, miR-148b plays a dual role in controlling EndoMT and angiogenesis, ultimately facilitating wound repair. Of note, while it may be relevant to EndoMT in SSc, direct evidence in SSc vasculopathy is lacking.

#### 4.1.6. miR-155

Another study demonstrates that miR-155 is a target of the angiotensin II type 1 receptor (AT1R), which is co-expressed in HUVECs and vascular smooth muscle cells (VSMCs) [156]. miR-155 represses AT1R expression, exerting a dual role in inflammation and vascular remodeling. It promotes HUVECs proliferation by modulating the expression of AT1R [156]. A silent polymorphism (+1166 A/C) in AT1R has been linked to cardiovascular diseases, likely through increased receptor activity. Notably, the +1166 C-allele disrupts base pairing in the AT1R 3′-UTR, weakening miR-155—mediated repression [156,157].

#### 4.1.7. let-7f and miR-27b

let 7f and miR-27b have been identified as essential players in EC-induced angiogenesis [134]. Inhibition of let-7f markedly reduced endothelial sprout formation *in vitro*, an effect associated with increased TSP-1 expression [43,80]. The findings are restricted to HUVECs; no *in vivo* SSc studies have been conducted.

#### 4.1.8. miR-152

Evidence indicates that miR-152 targets DNA methyltransferase 1 (DNMT1), thereby reducing global DNA methylation, including that of genes encoding bone morphogenic protein (BMP) receptor II and nitric oxide synthase (NOS) [78,144]. In SSc microvascular endothelial cells, downregulation of miR-152 promotes gene hypermethylation, leading to EC apoptosis, vasoconstriction, inflammatory cell recruitment, and subsequent fibroblast activation [144]. As patient-derived ECs studies are small-scale, clinical validation is required.

#### 4.1.9. miR-193b

Iwamoto et al. found that miR-193b targets urokinase-type plasminogen activator (uPA) [158], a molecule involved in vasculopathy that promotes neointimal growth, vascular remodeling, and fibrosis through enhanced ECM degradation [139]. In SSc dermal fibroblasts, miR-193b is downregulated [139], suggesting that it may contribute to proliferative vasculopathy [138]. Consistently, uPA levels were increased in SSc dermal fibroblasts following TGF-β stimulation [159]. Increased uPA signaling promotes cell proliferation and prevents apoptosis of human pulmonary artery smooth muscle cells through uPAR-independent pathways [159].

#### 4.1.10. miR-20a

Another miRNA, miR-20a, has been shown to target multiple genes within the TGF-β signaling pathway, including TGF-β type I receptor kinase (ALK5) and TGF-beta receptor II (TGF-βR2), thereby negatively regulating epithelial–mesenchymal transition (EMT) [112]. Data were derived from *in vitro* models; however, SSc-specific evidence is lacking.

#### 4.1.11. miR-146a

Studies have demonstrated that miR-146a influences cellular senescence by targeting the expression of nicotinamide adenine dinucleotide phosphate (NADPH) oxidase 4 (NOX4), an enzyme responsible for generating ROS through the reduction in molecular oxygen in the vessel wall [93,160]. It has no direct *in vivo* validation in SSc.

#### 4.1.12. miR-125a

miR-125a regulates both the pro-angiogenic and metabolic signaling pathways linked to dysfunctional angiogenesis. By modulating ECs metabolism and inhibiting 6-phosphofructo-2-kinase/fructose-2,6-biphosphatase 3 (PFKFB3), miR-125a reduces excessive vessel hyperbranching and promotes vascular normalization in response to pro-angiogenic stimuli, such as VEGF [135]. Since most evidence comes from tumor angiogenesis studies, the effect on SSc should be tested.

### 4.2. Anti-Angiogenic miRNAs

Several miRNAs inhibit angiogenesis by targeting pro-angiogenic factors or promoting ECs senescence, apoptosis, and inflammation.

#### 4.2.1. miR-221/222

Many miRNAs are involved in angiogenesis and are expressed in ECs [147]. According to Poliseno et al., miR-221 and miR-222 act as anti-angiogenic factors by suppressing the expression of the human protooncogene c-Kit receptor in ECs, thereby modulating the activity of stem cell factors, which are important regulators of angiogenesis and cell fate [90]. Overexpression of miR-221 and miR-222 in human umbilical vein endothelial cells (HUVECs) significantly reduces cell migration [90]. Moreover, miR-155, together with miR-221 and miR-222, has been shown to regulate Ets-1 transcription factor [156], which is induced by angiotensin II, TNF α, and thrombin and is essential for both inflammatory responses and microtubule formation [156]. miR-221 and miR-222 further modulate angiotensin II–mediated endothelial inflammation, whereas miR-155 regulates adhesion molecule expression [156]. Silencing of Dicer further revealed that miR-221/222 also regulate endothelial nitric oxide synthase (eNOS), influencing vascular remodeling, angiogenesis, and the mobilization and activity of stem and progenitor cells [161].

Recent findings suggest that miR-221/222 cluster significantly inhibits EC migration, tube formation, and wound healing *in vitro* despite exerting proliferative effects on VSMCs [33]. The recognition of the opposite effects of the miR-221/222 cluster in ECs and VSMCs highlights its importance in vascular remodeling and may open new therapeutic avenues to simultaneously limit neointimal proliferation while promoting reendothelialization [162]. Overexpression in HUVECs impairs wound healing and angiogenesis, while in SSc, their upregulation correlates with impaired angiogenesis in patient-derived ECs; therefore, patient validation should be extended.

#### 4.2.2. miR-92a

The miR-17~92 cluster encodes several miRNAs, including miR-17-5p, miR-17-3p, miR-18a, miR-19a, miR-20a, miR-19b-1, and miR-92-1, which induce tumor angiogenesis, promote cell proliferation, and suppress cancer cell apoptosis. Their pro-angiogenic activity is mediated in part by the suppression of predicted targets, such as Tsp1 and connective tissue growth factor (CTGF), thereby enhancing neovascularization [163]. Specifically, miR-18 downregulates CTGF expression, whereas miR-19 suppresses Tsp1 expression. Also, miR-17-5p regulates ECs’ proliferation and migration by targeting the anti-angiogenic factor tissue inhibitor of metalloproteinase 1 (TIMP-1) [43,94]. In contrast, miR-92a downregulates integrin (ITG) α5β1 (ITGα5) and Krüppel-like factors 4 and 5 (KLF4/KLF5), impairing angiogenic signaling and ECs survival [86,131,133,134]. Inhibition of miR-92a restores angiogenesis in ischemic models; however, there is currently no SSc-specific *in vivo* data.

#### 4.2.3. miR-15b, miR-16, miR-20a/b

Overexpression of miR-15b, miR-16, and miR-20 reduces the expression of VEGF, a critical regulator of angiogenesis in cancer models [88]. Their downregulation occurs under hypoxic conditions, likely mediated by the accumulation of the tumor suppressor p53 or stabilization of hypoxia-inducible factor 1α (HIF-1α) [43]. Thus, protein p53 (p53) may represent an important factor driving hypoxia-induced alterations in miRNA levels [88].

#### 4.2.4. miR-22

Yamakuchi et al. demonstrated that miR-22 modulates HIF-1α expression [117]. Overexpression of miR-22 suppresses HIF-1α, resulting in decreased VEGF production during hypoxia. Moreover, miR-22 affects ECs’ growth and invasion; therefore, it may be a potential anti-angiogenic molecule [117] Therefore, these findings should be validated in patients with SSc.

#### 4.2.5. miR-200c

miR-200c targets zinc finger E box binding homeobox 1 (ZEB1), which can be downregulated by ROS. Elevated expression of miR-200c influences the activation of the p53 and retinoblastoma protein tumor suppressor pathways, thereby promoting cellular senescence [93,143]. These data are restricted to *in vitro* assays.

#### 4.2.6. miR-34a

A recent study showed that miR-34a is expressed in primary ECs and its levels increase during cellular senescence. MiR-34a regulates EC proliferation and differentiation by downregulating Sirtuin 1 (SIRT1) [124], a longevity gene that protects cells against oxidative and genotoxic stress and modulates a number of proteins, including p53, heterodimer Ku (Ku70), NFκβ, and peroxisome proliferator-activated receptor gamma (PPAR-γ) [93]. Increased miR-34a level in ECs decreased SIRT1 and enhanced p53 acetylation, thereby promoting cellular senescence [124]. There are no *in vivo* SSc studies on it.

#### 4.2.7. miR-214

Mil et al. demonstrated that miR-214 is a novel modulator of angiogenesis in both *in vitro* and *in vivo* models. miR-214 directly targets the quaking homologue, KH domain RNA binding (QKI), and thereby suppresses expression and secretion of vascular growth factor [160]. No direct evidence was found for SSc.

#### 4.2.8. miR-217

MiR-217, which is expressed in young HUVECs and human coronary artery endothelial cells, promotes cellular senescence by inhibiting SIRT1 and consequently disrupting the SIRT1/forkhead box protein O1 (FOXO1) pathway [93,115].

#### 4.2.9. miR-328

miR-328 targets multiple adhesion molecules, including the hyaluronan receptor CD44 [164], which is involved in angiogenesis, wound healing, and leukocyte extravasation at the site of injury. Wang et al. show that elevated levels of miR-328 reduced the formation of capillaries, as well as cell adhesion and migration [164].

### 4.3. miRNAs Regulating EndoMT and EC Apoptosis

Several other miRNAs also play crucial roles in modulating EC plasticity and survival by influencing processes such as EndoMT and apoptosis. miR-21 and miR-200c have been implicated in TGF-β-induced EndoMT in cultured ECs, promoting fibrotic vascular remodeling in SSc [143,165,166]. In addition, miR-217 has been identified as a potent inducer of cellular senescence, primarily through the downregulation of SIRT1, which not only disrupts angiogenic signaling but also impairs vascular repair mechanisms [115]. In parallel, miR-152 targets DNMT1, contributing to epigenetic remodeling of ECs and promoting apoptotic pathways [144]. The collective actions of these miRNAs underscore a sophisticated regulatory network that balances vascular growth, repair, and senescence, ultimately shaping the angiogenic response. Their roles remain to be confirmed in disease-relevant animal models and well-powered patient cohorts.

Taken together, current evidence on angiogenesis-related miRNAs in SSc highlights both promising leads and significant gaps. Among the best-studied candidates, miR-126, miR-21, and miR-155 are consistently associated with vascular dysfunction, inflammation, and fibrosis in multiple SSc-derived models and small patient cohorts, strengthening their potential relevance as biomarkers or therapeutic targets. In contrast, other miRNAs, such as miR-130a, miR-210, miR-296, and miR-148b, are mainly supported by data from cancer biology, hypoxia, or developmental angiogenesis models, and their contribution to SSc remains speculative. Moreover, some inconsistencies between studies currently limit clinical translation and underscore the need for multicenter validation using standardized protocols. Furthermore, most mechanistic insights are derived from *in vitro* endothelial models or non-SSc disease contexts, which may not fully capture the complex immune-vascular-fibrotic interactions in SSc.

## 5. miRNAs as Diagnostic and Prognostic Biomarkers Results

miRNAs are differentially expressed in patients with SSc, suggesting vascular damage. Therefore, they may serve as biomarkers for diagnosis, molecular classification, and disease monitoring. The identification of reliable and minimally invasive biomarkers is the primary goal in the management of SSc, particularly for predicting vascular complications, assessing disease activity, and guiding treatment strategies. They can be determined in extracellular body fluids, such as serum, urine, saliva, and milk [167]. Serum miRNAs are found to be stable, reproducible, and consistent across individuals within a species [167,168]. Blood-based detection offers a minimally invasive, easily repeatable, and clinically feasible approach to monitor SSc. Current diagnostic methods rely on clinical evaluation and tissue biopsies, which are invasive, limited to local pathology, and often fail to capture systemic vascular and fibrotic changes. Thus, serum miRNAs allow for the early diagnosis, patient stratification, and longitudinal monitoring of vascular dysfunction and fibrosis. In addition, they are secreted into extracellular space by exosomes, microvesicles shed from cell membranes, encapsulated by damaged cells, or complexed with proteins (Ago2, Nucleophosmin (NPM1)) or lipoproteins (High-Density Lipoproteins (HDLs)), thereby protecting them from degradation by endogenous RNases and allowing their reliable detection in the blood, saliva, urine, and other body fluids. miRNAs are remarkably stable under various pH and temperature conditions [38,169,170,171].

Several studies have already highlighted the diagnostic or prognostic potential of individual miRNAs. Elevated levels of miR-142-3p have been proposed as a distinguishing marker of SSc compared with related autoimmune diseases [172].

Reduced miR-126 level has been linked to endothelial dysfunction and capillary rarefaction and is correlated with NVC abnormalities [148,173]. As it is essential for angiogenesis, vascular integrity, and endothelial cell migration, miR-126 downregulates proinflammatory cytokines and adhesion molecules like VCAM1 [148]. In cardiovascular pathology, circulating miR-126 concentrations are markedly reduced and are negatively correlated with pathological determinants [173]. Circulating miR-126 has diagnostic and prognostic value as a molecular marker of ischemic stroke, as well as a biomarker of disease severity in atrial fibrillation and heart failure [174,175].

In addition, decreased let-7a and let-7f levels are linked to increased collagen expression and impaired angiogenesis [176,177]. In contrast, increased levels of miR-21, miR-155, and miR-146a are associated with inflammation, fibrosis, and poor vascular regeneration [178,179]. Upregulation of miR-92a expression is inversely associated with angiogenic capacity and vessel density [180,181].

Specific vascular complications also appear to have distinct miRNA signatures. For example, patients with PAH often exhibit increased miR-210 and reduced miR-193b, whereas those with digital ulcers may show decreased miR-126 together with elevated miR-155, and increased levels of miR-21 and miR-22 have been linked to renal crisis [167,176,182,183,184]. These associations suggest that circulating miRNAs may help stratify patients by risk and guide early intervention. Dysregulation of the let-7 family, particularly let-7d and let-7b, has been correlated with elevated pulmonary arterial pressure, underscoring their potential as biomarkers for PAH in SSc [185].

A recent meta-analysis supports the idea that miRNA panels can differentiate between limited and diffuse SSc subtypes, predict organ involvement, and reflect responses to immunosuppressive or vasoactive therapies [186]. The potential angiogenic and anti-angiogenic miRNAs with diagnostic and/or prognostic value that are useful in clinical settings are presented in Table 2.

Overall, circulating miRNAs represent an emerging class of molecular indicators that can complement the current clinical and imaging tools. Larger multicenter studies with standardized protocols are needed to validate their utility; however, accumulating evidence supports their role in the disease classification, prognostication, and personalized management of systemic sclerosis.

## 6. Therapeutic Potential of Targeting miRNAs

The evolution of the microvasculature in patients with SSc plays a role in the prognosis of this disorder. Therefore, understanding the processes of angiogenesis and microvascular changes during the evolution of SSc is essential. Choosing the most effective treatment to achieve disease remission would be helpful. Dysregulated miRNA expression suggests their potential as therapeutic targets. Current research is evaluating the clinical application of miRNA-based therapies. Experimental strategies include exploring miRNA mimics to restore deficient pro-angiogenic miRNAs and miRNA inhibitors (antagomiRs) to block the pathological overexpression of anti-angiogenic or profibrotic miRNAs [187,188,189].

Downregulated miRNAs can be restored using miRNA mimics, for example, through adenoviral vectors carrying the target miRNAs [187]. Conversely, upregulated miRNAs may be silenced with miRNA inhibitors, such as antisense oligonucleotides (e.g., 2′-O-methyl, 2′-O-methoxyethyl or locked nucleic acid [LNA] oligonucleotides), which directly bind and block miRNA activity [187]. Although the specific inhibition or replacement of miRNAs can induce broad changes in gene expression, effective targeted therapies for SSc are still lacking. Further studies are needed to define viable therapeutic targets and clarify the precise roles of individual miRNAs before their clinical translation.

### 6.1. Experimental miRNA-Based Therapies in SSc

MiRNA mimics are synthetic oligonucleotides designed to restore the activity of downregulated miRNAs. In SSc, delivery of pro-angiogenic miRNAs, such as miR-126, let-7a, or miR-152, could theoretically improve endothelial repair and restore vascular homeostasis. Systemic administration of miRNAs has not yet been reported in skin diseases, including SSc. Preclinical models of other fibrotic or ischemic conditions have shown promising results, including enhanced angiogenesis, reduced apoptosis, and improved perfusion. Makino et al. reported that let-7a inhibits the expression of both α1 (I) and α2 (I) collagen, and its levels are downregulated in the affected skin of patients with SSc, as well as bleomycin-induced mouse fibrotic skin, a widely used model of skin fibrosis [190]. These findings suggest that let-7a may represent a potential therapeutic target. Indeed, restoration of let-7a expression in bleomycin-treated mice reduced type I collagen deposition and improved dermal architecture [190]. Similarly, miR-126 shows therapeutic potential by correcting impaired angiogenesis in SSc, leading to reversed endothelial dysfunction and improved vascular repair in ischemic models [173,175].

Treatment with miR-21 inhibitor suppresses TGF-β signaling, reduces fibrosis, and attenuates EndoMT in murine SSc models, whereas miR-155 inhibitor is linked with reduced inflammatory gene expression and vascular remodeling and dual effects on immune and vascular compartments [191,192,193]. Despite encouraging preclinical findings, no miRNA-based therapy has been approved for systemic sclerosis or other autoimmune fibrotic diseases.

The benefits of replacement therapy lie in its potential to normalize multiple dysregulated targets and pathways, offering a holistic approach compared to single-gene therapies. However, successful replacement requires effective delivery to target tissues, protection from nuclease degradation, and avoidance of immune activation. Off-target effects remain a major concern, as overexpression in non-diseased tissues could disrupt normal physiology.

### 6.2. Challenges in Delivery and Specificity

AntagomiRs and locked nucleic acid (LNA) inhibitors are used to silence overexpressed pathogenic miRNAs [194]. Therapeutic miRNAs are typically delivered using viral vectors (e.g., adenoviruses and lentiviruses), synthetic oligonucleotides (e.g., 2′-O-methyl and LNA-modified), or nanoparticle-based carriers (e.g., lipid nanoparticles and exosomes) [167,170,171]. Unlike conventional drugs that typically act on a single molecular target, miRNAs regulate networks of genes and pathways simultaneously [194]. This feature is particularly valuable in SSc, where vascular dysfunction, immune dysregulation, and fibrosis are interdependent processes. Modulating a single miRNA can therefore influence multiple pathogenic mechanisms in parallel, offering a more holistic approach to disease modification.

Another benefit is the capacity to restore physiological balance rather than simply block pathogenic signaling. For example, reintroduction of pro-angiogenic miRNAs (e.g., miR-126, let-7a, miR-152) could normalize endothelial repair and promote vascular regeneration, while inhibition of pathogenic miRNAs (e.g., miR-21, miR-155, miR-200c) could reduce inflammation as well as fibrosis.

In addition, miRNA therapeutics can also be helpful for precision medicine [195]. Circulating miRNAs are measurable in serum and plasma, making them potential candidates as non-invasive biomarkers. Therefore, the possibility of combining therapeutic and diagnostic approaches allows treatment to be personalized to the individual profile of miRNAs and disease activity [195,196].

Major challenges include achieving target specificity and efficient tissue delivery, especially for ECs in fibrotic organs, as well as minimizing off-target effects due to the pleiotropic nature of miRNAs [197]. The pleiotropic nature of individual miRNAs means that therapeutic inhibition or replacement can trigger widespread off-target effects. Additional concerns relate to immune system activation and toxicity associated with viral or nanoparticle delivery systems, as well as the need for precise dosing and biodistribution control. However, emerging technologies, such as hydrogel-based localized delivery, ECs-targeted aptamers, and miRNA-expressing mesenchymal stem cells, are being explored to overcome these barriers [197]. Moreover, the chemical modification of oligonucleotides introduces additional safety considerations. Therefore, although preclinical models support the potential of miRNA therapies, translation into the clinic requires careful optimization and rigorous evaluation.

### 6.3. miRNA–lncRNA–circRNA Interactions

miRNAs function within complex post-transcriptional regulatory networks. Notably, lncRNAs and circRNAs can act as miRNA sponges, sequester specific miRNAs, and diminish their availability to target [28,40]. For example, lncRNAs, such as HOTAIR and MALAT1, modulate angiogenesis and fibrosis through their regulation of miR-126 and miR-155 [198,199,200]. In contrast, circRNAs, such as circPTEN and circFOXO3, bind to miRNAs involved in ECs senescence and inflammatory responses [201,202]. Understanding these interactions may reveal new therapeutic targets and combination strategies for modulating the miRNA–ncRNA axis in SSc. Bartel’s early conceptual framework applied to miRNA biology underscores how such non-coding RNAs participate in competitive endogenous RNA (ceRNAs) networks, influencing miRNA availability and function within regulatory circuits [41,42]. Together, these interactions between miRNAs and ncRNA sponges may open novel avenues for combination therapies targeting the miRNA–ncRNA axis in SSc [28,42].

### 6.4. Clinical Translation: Current Status

Several miRNA-based therapeutics are currently being investigated in clinical trials for the treatment of various diseases. MRG-110 (miR-92a inhibitor) is in a Phase I trial for promoting vascular regeneration (NCT03603431) [203,204], while Remlarsen (miR-29 mimic) was tested in skin fibrosis as an anti-fibrotic agent (NCT02603224) [205], and Cobomarsen (miR-155 inhibitor), which was investigated in cutaneous T-cell lymphoma (NCT03713320) [206,207]. Although not yet applied to SSc, these trials provide proof of concept for miRNA modulation in fibrotic and vascular disease contexts.

## 7. Conclusions

Systemic sclerosis is a challenging disorder for clinicians owing to its variety of symptoms. This review summarizes the recent research progress in miRNA regulation of vascular pathology in patients with SSc. Because SSc progresses with severe manifestations, the most crucial challenge in research is the discovery of new biomarkers for the early diagnosis, prognosis, and prediction of treatment response.

MiRNAs have emerged as key regulators of vascular injury, fibrosis, and immune dysregulation in SSc. By influencing endothelial survival, angiogenesis, and fibroblast activity, they form an intricate network that links vascular damage with tissue scarring and organ dysfunction. Identifying the unique miRNA profile in patients with SSc would reveal its clinical relevance and could be useful for a better-individualized precision medicine strategy. Analyses of miRNA expression may be beneficial for the early diagnostic use and monitoring of current therapies.

Although several studies have demonstrated the involvement of specific miRNAs in regulating angiogenesis in SSc, several limitations must be acknowledged. First, most of the data were derived from small patient cohorts or *in vitro* models, which limit their generalizability and lack multicenter validation. Secondly, the heterogeneous nature of SSc and its clinical manifestations complicate the identification of consistent miRNA signatures. Moreover, variability in sample collection, RNA extraction, normalization methods, and standardization of miRNA detection methods remains challenging across studies. Addressing these limitations is essential for the clinical translation of miRNA-based diagnostics and therapeutics.

The development of miRNA-based diagnostics and therapeutics requires advances in delivery systems, specificity, and safety. Future studies should go beyond individual miRNAs to explore combinatorial miRNA effects, such as miR-126 and miR-130a together, miRNA–lncRNA–circRNA interactions, and non-canonical functions of miRNAs, such as nuclear localization and immune signaling. Longitudinal dynamics of miRNA expression during disease progression and treatment should be a point of interest as well.

Future research should aim to integrate miRNAs expression profiles with clinical phenotypes, such as diffuse and limited SSc, imaging biomarkers, autoantibody status, and organ-specific involvement, including the presence of PAH, interstitial lung disease, and renal crisis. In addition, combining miRNomics with other omics, such as transcriptomics, proteomics, epigenomics and metabolomics, may reveal the regulatory networks that drive SSc pathogenesis. Also, artificial intelligence (AI) models can be used to analyze complex datasets to identify novel miRNAs-based biomarkers and therapeutic targets. This integrative approach may enable early diagnosis, personalized response prediction, and treatment tailoring based on the miRNA signatures.

## Figures and Tables

**Table 1:** miRNA expression in vasculopathy in systemic sclerosis. Based on [91,92,93].

miRNAs	Level	Targets	Effects	Ref
Angiogenesis				
let-7f	↓	TIMP-1	Migration and proliferation of ECs, sprout formation	[94]
miR-1	↓	VEGF-A	Tube formation and proliferation of ECs	[95]
miR-10a	↑	MAP3K7, TAK1, βTRC	Modulation of proinflammatory EC phenotypes in atherosusceptible regions *in vivo*	[96]
miR-126	↑	VCAM-1 SPRED, PIK3R2, VEGFR-2, p85-β	Vascular integrity, cell adhesion and migration Proliferation of ECs Angiogenesis *in vivo*	[81,97,98]
miR-130a	↑	GAX, HOX-A5	Inhibition of ECs migration and proliferation	[82]
miR-133a/b	↑	TGF-β1	ECs’ proliferation and branch formation	[99,100]
miR-146a	↑	VEGF, PAK-1	Formation of new blood vessels	[101]
miR-155	↑	AT1R, VEGFR-2	Migration and proliferation of ECs Angiogenesis in the region of ischemia	[102,103,104,105]
miR-17	↓	ICAM-1, Janus Kinase 1	EC’s adhesion and migration	[106,107]
miR-17-5p	↑	TSP-1/CTGF, TIMP1	Migration and growth of ECs	[108]
miR-17~92	↑	TSP-1/CTGF	Migration and growth of ECs	[87,109]
miR-18a	↑	TSR/VEGFR-2	Migration and growth of ECs	[110]
miR-19a	↑	TSR/VEGFR-2	Migration and growth of ECs	[111]
miR-20a	↓ ↑	VEGF MKK3	Migration and growth of ECs ECs’ migration and angiogenesis	[112]
miR-21	↓	PTEN, SMAD7	ECs’ migration and proliferation	[113]
miR-210	↑	Ephrin-A3 NPTX1	ECs’ tube formation, proliferation, and migration ECs-mediated angiogenesis	[83,114]
miR-217	↑	FOXO, eNOS, SIRT1	Vessel formation, maturation	[115]
miR-218	↓	ROBO-1	Neovascularization Dysregulated endothelial migration Impaired retinal vasculature	[116]
miR-22	↑	HIF1α, VEGF	Inhibition of VEGF secretion	[117]
miR-221	↓	c-Kit, eNOS	Migration and proliferation of ECs Vessel permeability Tube formation, migration, and impaired wound healing	[118,119]
miR-222	↓	c-Kit, eNOS STAT5a	Migration and proliferation of ECs Vessel permeability Inflammation-mediated vascular remodeling Tube formation, migration, and impaired wound healing	[119,120]
miR-23a	↑	PHD1,2	Vascular permeability and cellular migration	[121]
miR-27b	↑	SEMA6A	Sprout formation	[122]
miR-296	↑	HGS	Tube formation and migration *in vitro*, angiogenesis *in vivo*	[84]
miR-31	↓	E-selectin	Immune cell infiltration at sites of inflammation	[123]
miR-34a	↑	SIRT1, p53	Angiogenesis blockade in ECs	[124,125,126,127]
miR-320	↓	IGF-1	Angiogenesis in diabetic ECs	[89]
miR-377	↓	CD133, VEGF	Angiogenesis	[128]
miR-378	↑	FUS-1, SUFU	Angiogenesis	[85]
miR-424	↑	CUL-2, HIF-1α	Cell proliferation, chemotaxis, angiogenesis, vascular remodeling	[129]
miR-4530	↑	VASH-1	Angiogenesis	[130]
miR-92a	↓	ITG-α5 KLF-4 KLF-5	Angiogenesis and vessel formation Proliferation Cell adhesion and cell interactions	[86,131,132,133,134]
Vascular inflammation				
miR-125a	↓	PFKFB3	ECs metabolism	[135]
miR-126	↑	SPRED-1 VCAM-1 ITG-α5	Inflammatory response Vascular integrity and homeostasis Angiogenesis	[73,81,98,136,137,138]
miR-193b	↓	PLAU	uPA signaling in MVECs	[139]
Cellular senescence				
miR-146a	↑	NOX4, KLF-4	Cell growth	[101]
miR-181a	↑	NOX4	Cell growth	[140,141,142]
miR-200c	↑	ZEB1	Cell growth	[143]
miR-217	↑	SIRT1, FOXO	Stress resistance	[115]
miR-34a	↓	SIRT1, p53	Stress resistance	[124,125]
miR-152	↓	DNMT1	Hypermethylation in MVECs	[144]
miR-30b	↓	PDGFRB	PDGF signaling	[145,146]

↑, increased; ↓, decreased; AT1R—angiotensin II type 1 receptor; CD133—prominin-1; COL1A1—collagen type 1, alpha 1; CTGF—connective tissue growth factor; CUL2—cullin-2; DNMT1—DNA methyltransferase 1; ECs—endothelial cells; eNOS—endothelial nitric oxide synthase; FBs—fibroblasts; FOXO—forkhead family of transcription factors; FUS-1—nuclear fusion protein; GAX—growth arrest-specific homeobox gene; HGS—hepatocyte growth factor-regulated tyrosine kinase substrate; HIF1α—hypoxia-inducible factor 1α; HOX-A5—homeobox protein HOX-A5; ICAM-1—intercellular adhesion molecule 1; IGF-1—insulin-like growth factor 1; ITG-α5—integrin α5; KLF-4, 5—Kruppel-like factor 4, 5; MAP3K7—mitogen-activated protein kinase 7; MKK3—mitogen-activated protein kinase 3; MMP1—matrix metalloproteinase 1; NOX4—NADPH oxidase 4; NPTX1—neuronal pentraxin 1; p53—protein p53; PAK1—serine/threonine-protein kinase; PDGFRB—platelet derived growth factor receptor beta; PFKFB3—6-phosphofructo-2-kinase/fructose-2,6-biphosphatase 3; PHD1,2—prolyl hydroxylase; PIK3R2—phosphoinositide-3-kinase regulatory subunit 2; PLAU—plasminogen activator, urokinase; PTEN—phosphatase and tensin homologue; Ref—reference; ROBO1—roundabout guidance receptor 1; SEMA6A—semaphoring 6A; SIRT1—sirtuin 1; SMAD3—mothers against decapentaplegic homologue 3; SMAD7—mothers against decapentaplegic homologue 7; SPRED—sprouty-related, EVH1 domain-containing protein 1; STAT5a—signal transducer and activator of transcription 5a; SUFU—suppressor of fused homologue; TAK1—transforming growth factor beta-activated kinase 1; TGF-β1—transforming growth factor beta-1; TIMP-1—tissue inhibitor of metalloproteinases; TSP1—thrombospondin 1; TSR—methyl-accepting chemotaxis protein; VASH1—vasohibin 1; VCAM-1—vascular cell adhesion protein 1; VEGF—vascular endothelial growth factor; VEGFR-2—vascular endothelial growth factor receptor-2; ZEB1—zinc finger E-box-binding homeobox 1; βTRC—β-transducin repeat-containing gene.

**Table 2:** Angiogenic and anti-angiogenic miRNAs in SSc.

miRNA	Direction of Change	Validated Targets or Pathways	Functional Role	Associated Vascular Complication	Diagnostic or Prognostic Potential
miR-126	↓	SPRED1, PIK3R2 (VEGF signaling)	Pro-angiogenic (lost in SSc)	Capillary loss, impaired angiogenesis	Circulating biomarker of SSc vasculopathy
miR-130a	↓	HOXA5, GAX	Pro-angiogenic (downregulated)	Defective neovascularization	Potential marker of angiogenesis defects
miR-210	↑	EFNA3, PHDs	Hypoxia-induced angiogenesis	Hypoxia-driven angiogenesis	Associated with hypoxia severity; candidate circulating biomarker
miR-132	↑	RAS/ERK pathway regulators	Pro-angiogenic, endothelial proliferation	Enhanced vascular repair	Exploratory biomarker (needs validation)
miR-92a	↑	ITGα5, KLF4/5	Anti-angiogenic	Inhibited angiogenesis, vascular rarefaction	Linked to digital ulcers; candidate biomarker
miR-155	↑	AT1R, VCAM-1	Pro-inflammatory, vascular injury	Inflammation-driven vasculopathy, PAH	Associated with PAH and inflammation severity
miR-29	↓	Collagens, ECM genes	Anti-fibrotic (loss promotes fibrosis)	Fibrosis, vascular remodeling	Circulating biomarker of fibrosis severity
miR-221	↑	c-Kit, endothelial proliferation	Anti-angiogenic, impairs endothelial repair	Endothelial dysfunction, impaired repair	Preliminary evidence in SSc; exploratory biomarker

↑, increased; ↓, decreased; AT1R—angiotensin II type 1 receptor; c-Kit (CD117)—stem cell factor receptor; ECM—extracellular matrix; EFNA3—ephrin-A3; GAX (MEOX2)—growth arrest-specific homeobox/mesenchyme homeobox 2; HOXA5—homeobox A5; ITGα5—integrin alpha-5; KLF4/5—Kruppel-like factor 4/5; PAH—pulmonary arterial hypertension; PHDs (EGLN1-3)—prolyl hydroxylase domain proteins; PIK3R2—phosphoinositide-3-kinase regulatory subunit 2; RAS/ERK pathway—rat sarcoma/extracellular signal-regulated kinase; SPRED1—sprouty-related EVH1 domain-containing protein 1; SSc—systemic sclerosis; VCAM-1—vascular cell adhesion molecule-1; VEGF—vascular endothelial growth factor.

## Data Availability

No new data were created.

## Abbreviations

The following abbreviations are used in this manuscript:

AECAs	Anti-endothelial cell antibodies
Ago2	Argonaut 2
AT1R	Angiotensin II type 1 receptor
CD133	Prominin-1
ceRNAs	Competing endogenous RNAs
CEUS	Contrast-enhanced ultrasound
circRNAs	Circular RNAs
c-Kit (CD117)	Stem cell factor receptor
COL1A1	Collagen type 1, alpha 1
CTGF	Connective tissue growth factor
CUL2	Cullin-2
DNMT1	DNA methyltransferase 1
ECM	Extracellular Matrix
ECs	Endothelial cells
EFNA3	Ephrin-A3
EndoMT	Endothelial-to-mesenchymal transition
eNOS	Endothelial nitric oxide synthase
EPCs	Endothelial progenitor cells
ET-1	Endothelin-1
FBs	Fibroblasts
FGF	Fibroblast growth factor
Fli1	Friend leukemia integration 1 transcription factor
FOXO	Forkhead family of transcription factors
Fra2	Fos-related antigen 2
FUS-1	Nuclear fusion protein
GAX	Growth arrest-specific homeobox gene
HGS	Hepatocyte growth factor-regulated tyrosine kinase substrate
HIF1α	Hypoxia-inducible factor 1alfa
HOX-A5	Homeobox protein HOX-A5
HUVECs	Human umbilical vein endothelial cells
ICAM-1	Intercellular adhesion molecule 1
IGF-1	Insulin-like growth factor 1
IL-1β	Interleukin 1beta
ITG-α5	Integrin alfa5
KLF-4, 5	Kruppel-like factor 4, 5
LNA	Locked nucleic acid
lncRNAs	Long non-coding RNAs
MAP3K7	Mitogen-activated protein kinase 7
MiRNAs	MicroRNAs
MKK3	Mitogen-activated protein kinase 3
MMP1	Matrix metalloproteinase 1
MVECs	Microvascular endothelial cells
NFκβ	Nuclear factor kappa beta
NO	Nitric oxide
NOX4	NADPH oxidase 4
NPM1	Nucleophosmin 1
NPTX1	Neuronal pentraxin 1
NVC	Nailfold videocapillaroscopy
OCTA	Optical coherence tomography angiography
p53	Protein p53
PAH	Pulmonary hypertension
PAK1	p21-Activated kinase 1
PDGF	Platelet derived growth factor
PDGFRB	Platelet derived growth factor receptor beta
PFKFB3	6-phosphofructo-2-kinase/fructose-2,6-biphosphatase 3
PHD1,2	Prolyl hydroxylase
PIK3R2	Phosphoinositide-3-kinase regulatory subunit 2
PLAU	Plasminogen activator, urokinase
Pre-miRNAs	Precursor miRNAs
Pri-miRNAs	Primary miRNAs
PTEN	Phosphatase and tensin homolog
RAS/ERK pathway	Rat Sarcoma/Extracellular Signal-Regulated Kinase
RISC	RNA-induced silencing complex
ROBO1	Roundabout guidance receptor 1
ROS	Reactive oxygen species
SEMA6A	Semaphoring 6A
siRNAs	Silent interfering RNAs
SIRT1	Sirtuin 1
SMAD3/7	Mothers against decapentaplegic homologue 3/7
SPRED	Sprouty-related, EVH1 do-main-containing protein 1
SSc	Systemic sclerosis
STAT5a	Signal transducer and activator of transcription 5a
SUFU	Suppressor of fused homolog
TAK1	Transforming growth factor beta-activated kinase 1
TGF-β1	Transforming growth factor beta-1
TIMP-1	Tissue inhibitor of metalloproteinases
TNF-α	Tumor necrosis factor alfa
TSP-1	Thrombospondin 1
TSR	Methyl-accepting chemotaxis protein
UTR	Untranslated region
VASH1	Vasohibin 1
VCAM-1	Vascular cell adhesion protein 1
VE-cadherin	Vascular endothelia cadherin
VEGF	Vascular endothelial growth factor
VEGFR-2	Vascular endothelial growth factor receptor 2
VSMCs	Vascular smooth muscle cells
XPO5	Exportin 5
ZEB1	Zinc finger E-box-binding homeobox 1
α-SMA	alfa-smooth muscle actin
β-TRC	beta-transducin repeat-containing gene
